# Long term (TEN YEARS) follow-up in a trembling patient with parkinsonian signs without dopaminergic denervation

**DOI:** 10.1016/j.prdoa.2025.100371

**Published:** 2025-07-21

**Authors:** Luca Angelini, Giulia Paparella, Petra Schwingenschuh, Rick C.G. Helmich, Matteo Bologna

**Affiliations:** a IRCCS Neuromed, Pozzilli, IS, Italy; b Neurophysiopathology Unit, Department of Translational Biomedicine and Neuroscience (DiBraiN), University of Bari Aldo Moro, Bari, Italy; c Department of Neurology, Medical University of Graz, Graz, Austria; d Centre for Cognitive Neuroimaging, Donders Institute for Brain, Cognition and Behaviour, Radboud University Nijmegen, Nijmegen, the Netherlands; e Department of Neurology, Center of Expertise for Parkinson and Movement Disorders, Donders Institute for Brain, Cognition and Behaviour, Radboud University Medical Centre, Nijmegen, the Netherlands; f Department of Human Neurosciences, Sapienza University of Rome, Italy

**Keywords:** Tremor, Parkinsonism, Sequence effect, Normal dopaminergic imaging, Case report

## Abstract

Some patients presenting with Parkinson’s disease (PD)-like features do not exhibit dopaminergic denervation, complicating clinical categorization. This case report describes a patient with a long-standing upper limb action tremor accompanied by parkinsonian signs, but without evidence of dopaminergic denervation, followed over an extended period of ten years. The case highlights the diagnostic challenges and underscores the importance of longitudinal observation in such atypical presentations.

A 76-year-old man with tremor and parkinsonian features was followed for ten years with clinical observation, video recordings, kinematic movement analysis, and structural and functional neuroimaging, including DaT-SPECT.

Clinical and kinematic evaluations revealed asymmetric upper limb action tremor, inconstant rest tremor, bradykinesia with sequence effect, re-emergent tremor, and progressive motor symptoms. Over time, tremor extended to cranial regions, and mild dystonic postures emerged. Structural neuroimaging ruled out secondary causes, and DaT-SPECT remained negative despite symptom progression. Dopaminergic therapy was ineffective, while propranolol and clonazepam provided partial relief.

This case highlights that parkinsonian motor features − such as re-emergent tremor and bradykinesia with sequence effect − can manifest and progress over time in patients with tremor, even in the absence of dopaminergic denervation. These findings underscore the need for further research into the role of the dopaminergic system in trembling patients. Moreover, they emphasize the importance of long-term follow-up and the integration of biomarkers to enhance the characterization and diagnostic accuracy of tremor syndromes.

Some patients with parkinsonian features may exhibit normal dopamine transporter imaging. These cases were previously classified as scans without evidence of dopaminergic deficit – SWEDD [1,2], even though this term is still debated.

Certain features, such as re-emergent tremor and bradykinesia with sequence effect, are considered specific to Parkinson’s disease (PD) [1], but clinical presentation in SWEDD can widely vary. In most cases, DaT-SPECT remains negative even after long-term follow-up. However, some cases show symptom progression and eventual conversion to PD [3], indicating a variable disease course.

We present a patient with slightly progressive parkinsonian signs − including re-emergent tremor and a bradykinesia with sequence effect − without evidence of dopaminergic denervation over a decade of follow-up.

A 76-year-old man has been followed at the Movement Disorders Clinic of Sapienza University of Rome for asymmetric upper limb tremor and additional parkinsonian signs with slight progressive course. Clinical evaluations included standardized rating scales and video recordings. Kinematic analysis using an optoelectronic system was performed to objectively assess tremor and bradykinesia (details in Supplementary Material). Radiological follow-up comprised brain MRI, and repeated semiquantitative DaT-SPECT to monitor potential changes in dopaminergic function.

The patient was born to non-consanguineous parents. His father had developed a bilateral upper limb action tremor in his seventies. Medical history included hypertension, hypercholesterolemia, and scoliosis. Action tremor began at age 60 in the upper limbs, predominantly on the right side, and has gradually worsened. The tremor moderately impacts daily activities and does not improve with alcohol consumption. The patient has also reported mild motor slowing over the past five years and mild insomnia but denies prodromal PD symptoms such as hyposmia, constipation, or REM sleep behavior disorder.

Physical examinations conducted in 2017, 2019, and 2024 are documented in the accompanying videos (Supplementary Material). The initial examination revealed bilateral postural upper limb tremor, predominantly on the right and most evident in the wing-beating position, along with re-emergent tremor, mild kinetic tremor, and an inconstant unilateral rest tremor observed when the right limb was resting on the lap or armrests. There was slight slowing of finger tapping with hesitations and hypomimia. Additionally, the patient exhibited a mild rightward head tilt and head tremor. Over time, the latency of the postural tremor disappeared, kinetic tremor worsened (Fig. 1A), and mild resting tremor and movement slowness became more apparent in the left arm. Repetitive upper limb movements showed a mild decrement in velocity and amplitude (sequence effect) in the left hand, which progressed and eventually involved the right hand. In the last evaluation, mild bradykinesia was also observed in the repetitive movements of the legs (Supplementary Video). Handwriting analysis demonstrated worsening tremor without micrographia (Fig. 1A). The patient also developed voice, chin, and perioral tremor; dystonic postures of the upper limbs characterized by arm adduction, wrist flexion, and ulnar deviation of the third to fifth fingers, which were more pronounced in the left upper limb, task-specific, not reported by the patient as painful, and with no alleviating maneuvers observed; and reduced left arm swing. The patient exhibited neither rigidity nor postural instability. No inconsistent or incongruent tremor features were observed. The remainder of the neurological examination was unremarkable. The Fahn-Tolosa-Marin Tremor Rating Scale score increased from 44 in 2017 to 57 in 2024, while the MoCA score remained stable at 28.

**Fig. 1:**
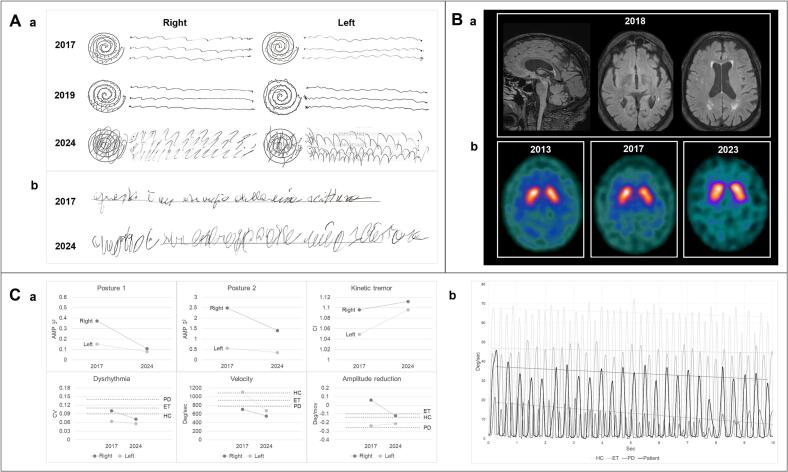
Clinical, neuroimaging and kinematic data. A: Clinical data. a: Archimedes spirals and line drawings performed with both hands across three evaluations. b: Handwriting samples from the first and last evaluation. B: MRI and DaT-SPECT data. a: MRI FLAIR sequence images from 2018 showing mild white matter hyperintensities of presumed vascular origin. b: DaT-SPECT images from 2013, 2017 and 2023 displaying normal radioligand uptake. Striatum-to-occipital uptake ratios were 3.56 in 2013, 3.89 in 2017, and 3.48 in 2023, with asymmetries of 3.8% on the right, 0.2% on the left, and 6.1% on the right, respectively. These findings indicate stable and symmetrical striatal dopaminergic innervation. C: Kinematic data of tremor and bradikinesia. a: Evolution of kinematic data for tremor in different conditions (top panels) and bradykinesia during the finger-tapping task (bottom panels). Posture 1: arms outstretched in front of the chest with hands pronated. Posture 2: arms flexed at the elbows with palms facing downward. AMP: tremor amplitude expressed in m/s^2^ RMS; CI: curvature index. CV: coefficient of variation. The dashed lines represent the cross-sectional mean values of the different parameters in 158 healthy controls (HC), 82 ET, and 192 PD patients. Details on the various kinematic measures and mean values shown are detailed in the Supplementary Material. Notably, kinetic tremor and bradykinesia worsened over time, while the amplitude of postural tremor decreased over time; resting tremor was not captured by kinematic assessment because of its inconstancy. b: Example of the kinematic traces of finger-tapping movement amplitude, expressed in degrees per second, comparing the patient (black line) with a representative healthy control (HC), ET, and PD patient (light grey, medium grey, and dark grey lines, respectively). The dashed lines represent the trend lines of movement amplitude, passing through the maximum amplitude peak points. The patient's trace shows a progressive reduction in movement amplitude.

Kinematic data from this patient were compared with those of 158 healthy controls, 82 patients with essential tremor (ET), and 192 patients with PD from our datasets (details in Supplementary Material). The patient showed a worsening of kinetic tremor, accompanied by a reduction in postural tremor. Finger-tapping kinematics revealed reduced velocity, which further declined over time. A sequence effect was observed in the left hand (Fig. 1C).

Brain MRI revealed stable mild subcortical and periventricular white matter hyperintensities of vascular origin, with no other notable findings. DaT-SPECT scans in 2013, 2017, 2019, and 2023 showed normal results (Fig. 1B). Furthermore, in 2024 the patient underwent next-generation sequencing targeting a panel of genes associated with hereditary parkinsonism, which yielded negative results.

Sequential trials with rasagiline, levodopa/carbidopa, and pramipexole MR were initiated since 2013 without improvement. The patient was also treated with propranolol (60 mg daily), which led to subjective tremor improvement; however, bradycardia limited further dose increases. Clonazepam (1.5 mg daily) was subsequently added, providing additional benefit.

This case report describes a clinical and radiological follow-up of more than ten years in a trembling patient with parkinsonian features, including re-emergent tremor and bradykinesia with sequence effect. Despite the progressive clinical picture, serial imaging consistently demonstrated preservation of the nigrostriatal dopaminergic system. Furthermore, kinematic analysis provided an objective and quantitative assessment of motor parameters. Notably, the presence and progression of the sequence effect is a new observation, and the presence of re-emergent tremor reinforce previous but scarce evidence. Furthermore, the use of kinematic analysis, compared against a large database of healthy controls, ET, and PD patients, allowed for precise quantification and differentiation of motor abnormalities, reinforcing the strength and novelty of our observation.

Contrary to prior findings [1,2], our patient exhibited a sequence effect, suggesting this phenomenon is not exclusive to PD. While re-emergent tremor is considered characteristic of PD [1,2], our case also demonstrated a distinct latency in postural tremor exceeding 3 seconds, particularly in the first evaluation, as described in earlier studies [2,4]. Additionally, hypomimia, another typical PD feature, was observed, consistent with previous findings [2]. Taken together, these observations illustrate how clinical boundaries between diagnostic categories can be more nuanced than previously assumed. They underscore the importance of precise clinical phenotyping, ideally supported by objective measurements and longitudinal follow-up to capture potential disease evolution.

The lack of response to dopaminergic therapy is consistent with previous evidence showing absent, uncertain, or unsustained treatment response in the majority of SWEDD cases where such therapy was attempted [5]. This underscores the importance of accurate diagnosis to avoid ineffective treatments and minimize the risk of adverse effects in patients who do not benefit from dopaminergic medications.

The progressive worsening of finger-tapping suggests parkinsonian signs can slowly advance without dopaminergic loss. Although subtle basal ganglia degeneration cannot be entirely excluded, long follow-up makes it less likely, supporting extranigral pathways’ involvement. Thus, relying on DaT-SPECT alone is insufficient, highlighting the need for additional functional imaging targeting non-dopaminergic systems to better elucidate the pathophysiology of parkinsonian signs in patients without PD.

This case also illustrates the practical challenges neurologists face when managing patients with overlapping parkinsonian and tremor features but normal DaT-SPECT imaging. In such scenarios, diagnostic categorization is difficult, particularly at the first visit, making longitudinal follow-up essential. Decisions regarding repeat imaging, additional diagnostic investigations (including genetic testing and biomarkers of non-dopaminergic systems), and therapeutic strategies are complex and must be tailored to the individual case until more definitive evidence becomes available. Treatment should primarily be guided by symptom burden and patient needs. A limited trial of levodopa may be reasonable; however, in the absence of clear benefit, discontinuation is advised to avoid unnecessary exposure and potential side effects.

In this case, dystonic tremor is the most likely diagnosis due to tremor asymmetry, posture dependence, subtle dystonic features, and reduced arm swing. However, the lack of overt dystonia complicates the diagnosis according to current MDS criteria, which require clear dystonic posturing. Bradykinesia with sequence effect is also atypical for dystonic tremor [6]. Although resembling PD, normal DaT-SPECT, the absence of prodromal symptoms, and the lack of response to dopaminergic therapy argue against it. ET plus is another possibility, given the action tremor, slow course, and inheritance, but tremor asymmetry and parkinsonian features make this diagnosis less likely. Among other differential diagnoses, the lack of incongruent features, distractibility, or entrainment, and the progressive nature of symptoms over a decade, argue against a functional origin. Vascular parkinsonism is also considered unlikely due to the absence of significant radiological burden beyond mild chronic microvascular changes.

In conclusion, some tremor patients may show PD-like features despite divergent clinical and radiological findings, complicating diagnosis. Recognizing these cases is essential for accurate assessment. Extended follow-up and biomarkers may improve diagnostic precision and deepen understanding of underlying pathophysiology beyond strict diagnostic categories.

## Financial disclosures

This work was supported by the Italian Ministry of Health (Current Research 2025). The authors declare that there are no conflicts of interest relevant to this work. The authors declare that there are no additional disclosures to report.

## Ethics statement

The authors confirm that approval of an institutional review board was not required for this study. Written informed consent was obtained from the patient for the publication of his data and the online distribution of the related video material.

## CRediT authorship contribution statement

**Luca Angelini:** Writing – original draft, Software, Investigation, Formal analysis, Data curation. **Giulia Paparella:** Writing – review & editing, Project administration, Investigation. **Petra Schwingenschuh:** Writing – review & editing. **Rick C.G. Helmich:** Writing – review & editing. **Matteo Bologna:** Writing – review & editing, Supervision, Conceptualization.

## Declaration of competing interest

The authors declare that they have no known competing financial interests or personal relationships that could have appeared to influence the work reported in this paper.

## Appendix A Supplementary data

Supplementary video 1 The clinical evaluation of the patient in 2017, 2019, and 2024 is shown in the three segments of the video. Each segment includes the patient’s assessment with arms at rest, during the maintenance of two postures, the finger-to-nose test, and finger tapping task. The 2024 segment additionally includes sustained phonation to assess vocal tremor, voluntary head and facial movements, and gait evaluation.

Supplementary video 2 The supplementary video shows five standardized motor tasks — finger tapping, hand movements, pronation–supination of the hands, toe tapping, and leg agility — performed according to MDS-UPDRS Part III during the 2024 evaluation.
