# Supplementary material for: Long term (TEN YEARS) follow-up in a trembling patient with parkinsonian signs without dopaminergic denervation
Source: Clin Park Relat Disord. 2025 Jul 21;13:100371. doi: 10.1016/j.prdoa.2025.100371 (PMC12311531; doi:10.1016/j.prdoa.2025.100371)
Supplement: Supplementary Data 1 [file mmc3.docx]

**SUPPLEMENTARY METHODS**

We performed kinematic analysis using an optoelectronic motion capture system. For postural tremor, acceleration signals were processed using a Fast Fourier Transform (FFT) to identify the individual peak frequency. Tremor amplitude was then quantified by calculating the root mean square (RMS) of the acceleration traces within a ±1 Hz window centered on this peak, and expressed in m/s² RMS. For kinetic tremor, an algorithm was applied to compute the Curvature Index (CI) as an indirect measure of tremor amplitude. CI was defined as the ratio between the total path length of the arm trajectory and the straight-line distance between the initial and final positions. Higher CI values indicate more irregular and curved trajectories, reflecting greater kinetic tremor amplitude.

Bradykinesia was assessed using a repetitive finger-tapping task involving the index finger and thumb. Each side was tested in three 15-second trials. Movement velocity was computed using a linear regression model applied to the angular displacement data over time, and expressed in degrees per second (deg/s). To evaluate the sequence effect, the slope of movement amplitude across successive taps was calculated (in degrees per movement, deg/mov); negative slope values indicated progressive amplitude reduction. Dysrhythmia was assessed via the coefficient of variation (CV), defined as the ratio of the standard deviation to the mean of inter-tap intervals, with higher CV values reflecting greater irregularity in timing.

**SUPPLEMENTARY RESULTS**

The kinematic findings of the present case were compared to the data from a reference sample consisting of healthy controls (HC), patients with essential tremor (ET), and patients with Parkinson’s disease (PD), all assessed using the same kinematic protocol described in the methods.

In this reference sample, the mean ± standard deviation for dysrhythmia (CV) was 0.09 ± 0.04 in HC, 0.11 ± 0.04 in ET, and 0.14 ± 0.07 in PD. Movement velocity was 1087.16 ± 252.16 degrees per second in HC, 914.77 ± 292.38 in ET, and 778.38 ± 279.26 in PD. Amplitude reduction, measured as the slope of movement amplitude (deg/mov), was –0.14 ± 0.21 in HC, –0.10 ± 0.20 in ET, and –0.26 ± 0.31 in PD.

These values provide a normative framework for interpreting the patient's motor performance in relation to dysrhythmia, movement speed, and sequence effect.
